# Mechanistic blockade of RSV infection by a pre-F targeting single-domain antibody-Fc fusion protein

**DOI:** 10.1128/aac.00541-26

**Published:** 2026-09-16

**Authors:** Shuang Liu, Xiran Qiu, Shiyu Guo, Liying Wang, Hui Shen, Maomao An

**Affiliations:** 1 Department of Pharmacology, Shanghai Tenth People’s Hospital, Tongji University School of Medicine 278245, Shanghai, China; 2 Research and Technology Development Center, Starmab Biologics (Suzhou) Co., Ltd., Shanghai, China; 3 Department of Clinical Laboratory Medicine, Shanghai Tenth People’s Hospital, Tongji University School of Medicine 278245, Shanghai, China; Chinese Academy of Medical Sciences & Peking Union Medical College, Beijing, China

**Keywords:** RSV, single domain antibody, Fc-fusion protein, conformational change, infection

## Abstract

Respiratory syncytial virus (RSV) is a primary cause of acute lower respiratory tract infections in children and the elderly. Despite its clinical impact, effective prophylactic options for RSV remain limited. The prefusion conformation of the RSV fusion (F) protein is a critical target for neutralizing antibodies; however, the specific roles of prefusion versus postfusion states in triggering inflammatory responses have remained unclear. We report the development of Hu3F5, a novel prefusion F-specific single-domain antibody-Fc fusion protein. Hu3F5 demonstrates exceptional neutralizing activity *in vitro* (IC_50_: 0.58–9.15 ng/mL), potent *in vivo* efficacy, and a favorable safety profile in non-human primates. Crucially, our study reveals that the postfusion conformation, rather than the prefusion state, induces IL-6 elevation. Mechanistic analyses show that Hu3F5 neutralizes RSV by locking the F protein in its prefusion state, independent of Fc-mediated effector functions. This physical stabilization prevents the transition to the postfusion conformation, thereby blocking membrane fusion and subsequent inflammatory signaling, as well as the formation of cytopathic syncytia. By identifying the postfusion state as the driver of RSV-induced inflammation, this study positions Hu3F5 as a promising clinical candidate for prophylaxis. Furthermore, it provides new insights into RSV pathogenesis and the efficacy of “locking” mechanisms in viral neutralization.

## INTRODUCTION

Respiratory syncytial virus (RSV) was discovered over six decades ago (1–3) and remains one of the leading global causes of severe lower respiratory tract infections (4, 5), especially in children under five, adults over 65, and individuals with underlying medical conditions (4). The global health burden is substantial; for instance, RSV caused an estimated 101,400 deaths in children under five worldwide (6). Among older adults (≥65 years) in high-income countries, it is responsible for approximately 1.5 million acute respiratory infection events and 14,000 in-hospital deaths annually (7). Furthermore, the epidemiological data on RSV from low- and middle-income countries are scarce due to limited healthcare (8), which means that the burden of RSV infection may be underestimated.

On the surface of the RSV, there is an important glycoprotein—the fusion protein—which mediates the critical virus-cell membrane fusion process and is highly conserved across RSV strains (1, 9, 10). It has become a primary target for current anti-RSV drug development. When RSV attaches to the host cell membrane, the F protein is triggered to undergo an abrupt conformational transition from the prefusion state to the postfusion state; this change promotes the exposure of the fusion peptide. Subsequently, the exposed peptide inserts into the host cell membrane and drives membrane fusion, thereby facilitating the entry of the RSV genome into cells (11–14). Furthermore, the F protein has also been shown to contribute to the inflammatory response caused by RSV and is a major factor in RSV-induced bronchiolitis and mortality (15, 16). However, given that the F protein exists in two conformational states, which state of the F protein is responsible for triggering the inflammatory response remains unclear.

Despite decades of research, developing effective interventions for RSV remains challenging, particularly in achieving robust protection without eliciting aberrant immune responses (6). For active immunization in older adults, three vaccines have recently been approved for clinical use (17–19). For infants, no active vaccine is yet available, and current options are limited to passive immunoprophylaxis with monoclonal antibodies, which continue to face challenges posed by resistant viral strains (20–24). Consequently, there remains a significant unmet need worldwide for broadly protective and durable RSV prevention strategies, particularly for the pediatric population.

Herein, we identified a novel anti-RSV single-domain antibody-Fc fusion protein, Hu3F5, which exhibits highly potent *in vivo* and *in vitro* efficacy against RSV infection. Furthermore, our study clarified that the postfusion conformation of the F protein is the structural state responsible for triggering the inflammatory response. Further investigation revealed that Hu3F5 exerts its potent neutralizing activity precisely by locking the F protein in its prefusion state. In conclusion, our results provide a clearer explanation for the enhanced effectiveness of antibodies that target the prefusion F protein, thereby contributing new theoretical perspectives on the mechanism of RSV infection.

## RESULTS

### Screening and identification of pre-F-targeting neutralizing single-domain antibody-Fc fusion proteins

Given that antibodies directed exclusively against the prefusion conformation (pre-F) possess significantly enhanced neutralizing potency relative to those targeting the postfusion form (post-F) or exhibiting cross-reactivity to both conformations (25), we focused on isolating single-domain antibodies (VHHs) specific for pre-F from immunized alpacas. A high-purity pre-F protein was produced and used as the immunogen for alpaca immunization (Fig. S1A). We confirmed that the purified protein specifically bound to nirsevimab, a known positive-control antibody (Fig. S1B), which verified that it retained the correct native conformation. Following sequencing, we identified unique VHH clones and engineered them into Fc-fusion proteins by fusing them with an IgG1 Fc domain.

The binding specificity of the recombinant Fc-fusion proteins was characterized using enzyme-linked immunosorbent assay (ELISA). We identified 12 clones that specifically bound to pre-F with half-maximal effective concentrations (EC₅₀) ranging from 3.93 to 15.12 ng/mL and showed low or negligible binding to post-F (Fig. S2). Bio-layer interferometry (BLI) confirmed that all 12 Fc-fusion proteins exhibited high affinity for pre-F, with equilibrium dissociation constants (KD) ≤0.34 nM (Fig. S3; Table S1).

Among these 12 pre-F-specific Fc-fusion proteins, five neutralized the live RSV Long strain *in vitro*, with half-maximal inhibitory concentrations (IC₅₀) ranging from 16.23 to 126.7 ng/mL. The remaining seven clones showed no neutralizing activity under the tested conditions (Fig. S4).

### Hu3F5 demonstrates the most potent broad-spectrum neutralizing activity

**Fig 1:**
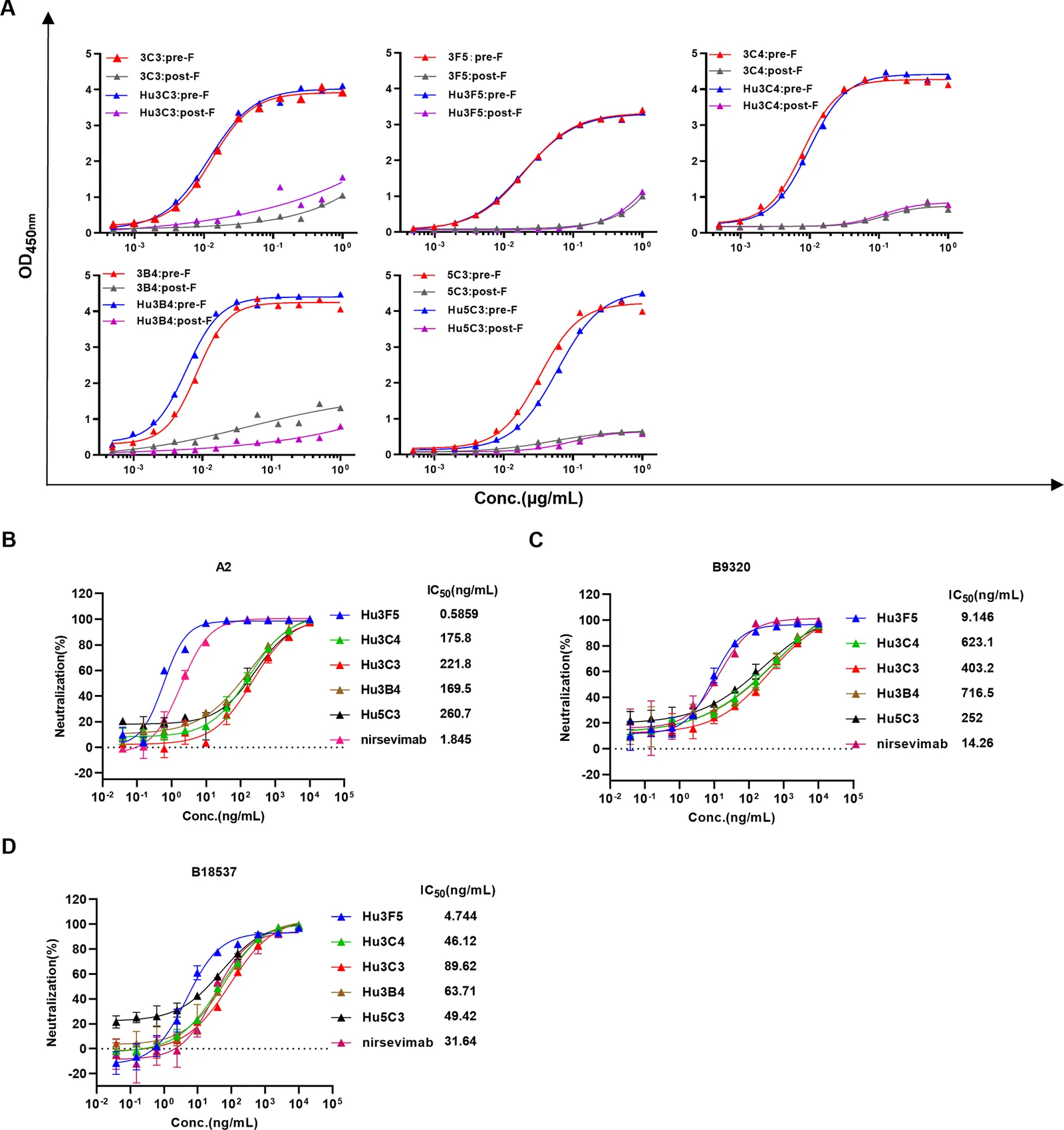
Hu3F5 demonstrates the most potent broad-spectrum neutralizing activity. (**A**) Binding activity of humanized Fc-fusion proteins was measured by ELISA. Data are representative of one of three independent experiments. (**B–D**) Neutralizing activities of the five humanized Fc-fusion proteins against RSV A2 (**B**), RSV B9320 (**C**), and RSV B18537 (**D**) were measured with a live RSV neutralization assay. Nirsevimab was used as a positive control. Data are representative of one of three independent experiments and are presented as mean ± SD (*n* = 2) (**B–D**). ELISA, enzyme-linked immunosorbent assay; IC_50_, half-maximal inhibitory concentration.

We humanized five candidate molecules that exhibited neutralizing activity against the RSV Long strain. This was achieved by introducing amino acid mutations into the framework regions of their variable domains based on the human germline genes with the highest homology, generating five humanized Fc-fusion proteins designated as Hu3C3, Hu3C4, Hu5C3, Hu3B4, and Hu3F5. Binding analysis demonstrated that all humanized variants retained binding activity comparable to that of their camelid counterparts (Fig. 1A).

The neutralization potency of the five humanized Fc-fusion proteins was evaluated against representative laboratory strains of RSV subgroups A and B (A2, B9320, and B18537) using live virus neutralization assays. Among them, Hu3F5 exhibited the most potent neutralizing activity against three strains, with IC₅₀ values ranging from 0.5859 to 9.146 ng/mL. Furthermore, Hu3F5 showed markedly superior potency compared to the other four candidates, with potencies that were >289-fold higher against RSV A2, >27-fold higher against RSV B9320, and >9.7-fold higher against RSV B18537 (Fig. 1B through D).

### Hu3F5 exerts potent neutralizing activity through a unique binding epitope

**Fig 2:**
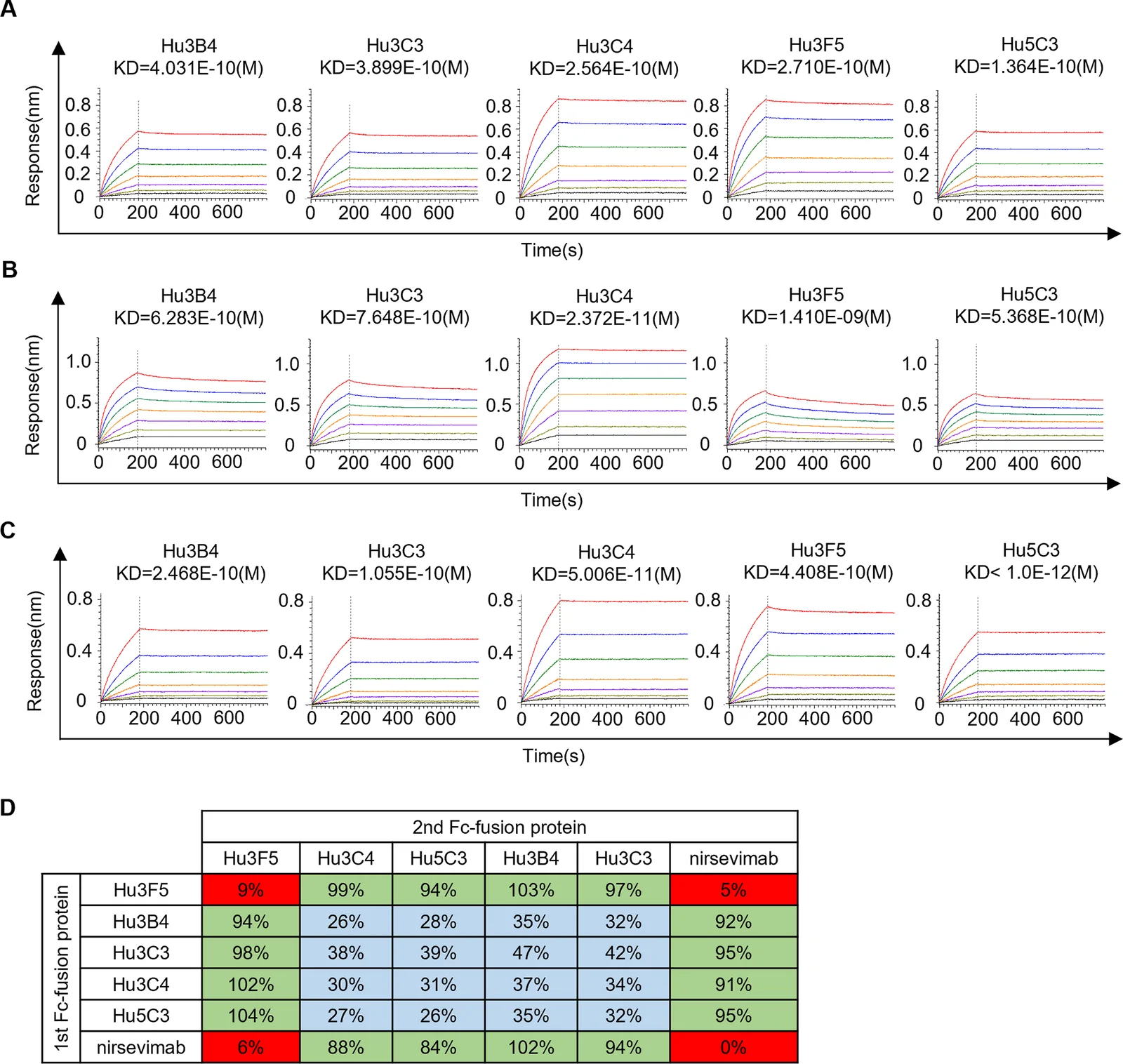
Hu3F5 exerts potent neutralizing activity through a unique binding epitope. (**A–C**) Kinetic binding curves of five humanized Fc-fusion proteins to RSV A2-pre-F (**A**), RSV B9320-pre-F (**B**), and RSV B18537-pre-F (**C**) as determined by BLI. (**D**) Epitope binning analysis via BLI-based cross-competition assay. Humanized Fc-fusion proteins are grouped into distinct bins (color-coded). Red shading indicates full competition between the first and second humanized Fc-fusion proteins; blue shading denotes partial competition; green shading indicates no competition. Data are representative of one of three independent experiments (**A–D**); BLI, bio-layer interferometry; *K*_*D*_, equilibrium dissociation constant.

To investigate the mechanism by which Hu3F5 exhibits the most potent neutralization against three RSV strains of different subtypes, we further characterized the binding properties of the five humanized Fc-fusion proteins. The *K*_*D*_ and EC_50_ values for their binding to three pre-F proteins were determined using BLI and ELISA, respectively. Specifically, the *K*_*D*_ values for Hu3F5 binding to the three pre-F proteins ranged from 0.2710 to 1.410 nM. Similarly, high-affinity binding was observed for Hu3B4 (*K*_*D*_: 0.2468–0.6383 nM), Hu3C3 (*K*_*D*_: 0.1055–0.7648 nM), and Hu3C4 (*K*_*D*_: 0.05006–0.2564 nM). For Hu5C3, the *K*_*D*_ values against all three pre-F proteins were ≤0.5368 nM (Fig. 2A through C). The EC₅₀ values for pre-F binding fell within a narrow and comparable range for Hu3B4 (EC_50_: 75.72–106.1 ng/mL), Hu3C3 (EC_50_: 72.5–101.5 ng/mL), Hu3C4 (EC_50_: 74.57–94.26 ng/mL), and Hu5C3 (EC_50_: 74.14–91.77 ng/mL), whereas Hu3F5 displayed a wider range of 51.46–809.5 ng/mL (Fig. S5).

We found that Hu3F5 did not exhibit the strongest binding affinity for the pre-F protein at the molecular level. This observation is similar to the findings of Ku et al. (26). In their study on SARS-CoV-2 antibodies, they demonstrated no correlation between the strength of neutralizing activity and the level of binding affinity to the antigenic protein. Furthermore, it has been established that antigenic site Ø, located exclusively at the apex of the pre-F trimer, can elicit the most potent neutralizing activity (11). Therefore, we performed epitope binning experiments among the five Fc-fusion proteins using BLI, with nirsevimab as a control antibody targeting site Ø. The BLI-based cross-competition assay revealed that the five candidates segregated into two distinct clusters: Hu3F5, which fully competed with nirsevimab for site Ø, and the remaining four Fc-fusion proteins, which targeted an epitope entirely distinct from that of Hu3F5. Furthermore, among the four non-Hu3F5 candidates (Hu3B4, Hu3C3, Hu3C4, and Hu5C3), each exhibited mutual competition, indicating that they all bind to a shared epitope different from site Ø (Fig. 2D). Therefore, we preliminarily conclude that the potent neutralizing activity of Hu3F5 may be mechanistically attributed to its unique engagement of site Ø—an epitope distinct from that targeted by the other four antibodies.

### Prophylactic efficacy of Hu3F5 in RSV-infected mice

**Fig 3:**
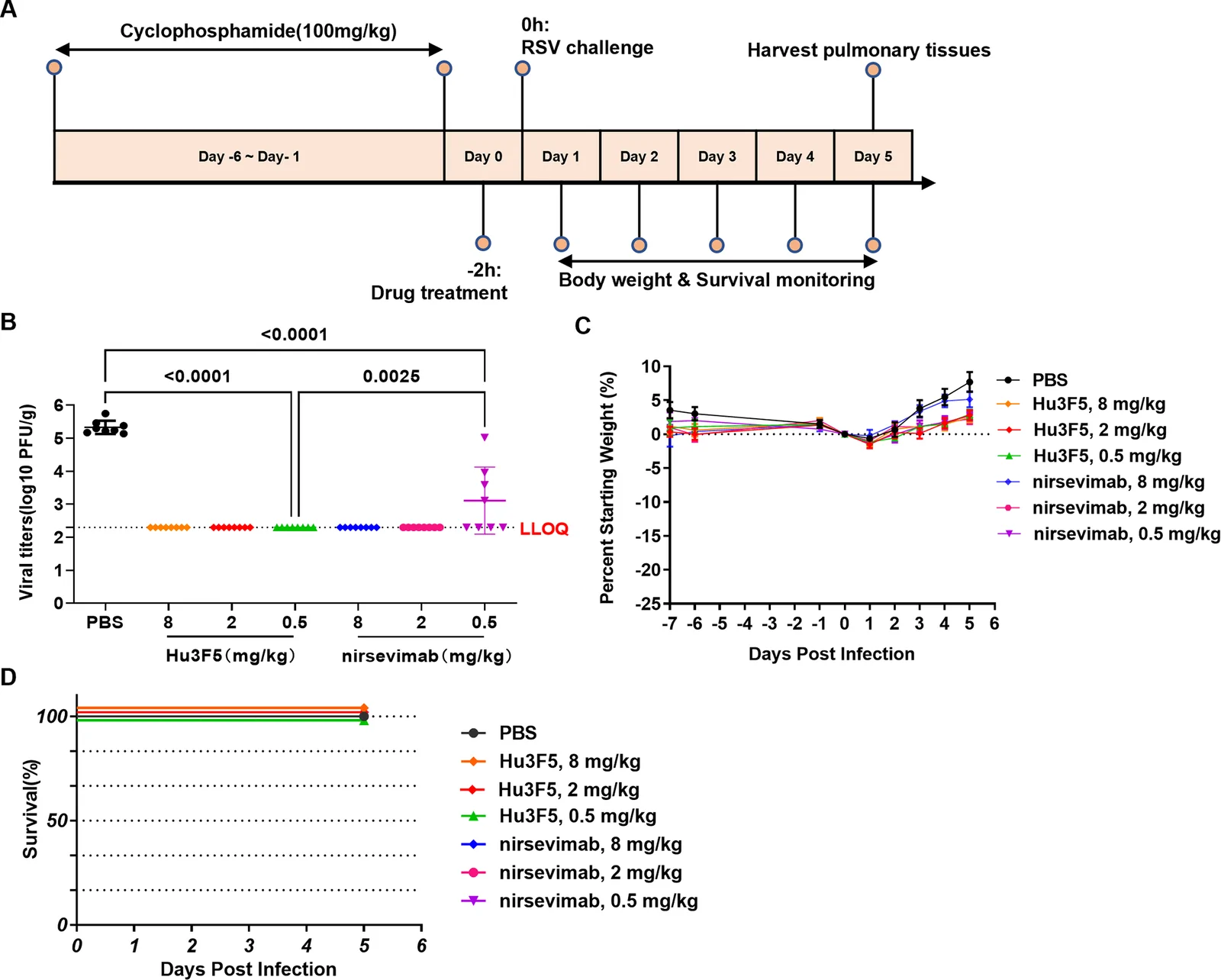
Prophylactic efficacy of Hu3F5 against RSV A2 infection in mice. (**A**) Schematic diagram of RSV prophylactic efficacy. Immunosuppressed BALB/c mice received a single intravenous injection of either Hu3F5 or nirsevimab at three dosage levels (8 mg/kg, 2 mg/kg, and 0.5 mg/kg) on day 0, 2 hours before challenge with 1 × 10⁵ PFU of RSV A2. PBS served as the negative control. Lung tissues from each group were harvested on day 5 post-infection. (**B**) RSV lung viral titers in mice 5 days post-infection. Error bars represent mean ± SD (*n* = 8). The dotted line indicates the limit of detection (LLOQ). Statistical significance was determined by one-way ANOVA with Tukey’s multiple comparisons test. ****, *P* < 0.0001; ***, *P* < 0.001; *, *P* < 0.05; ns, *P* > 0.05. (**C**) Body weight monitoring of RSV-infected mice. Body weights were recorded daily for 5 days post-infection. Data are presented as mean ± SEM from three independent experiments (*n* = 8). (**D**) Survival curves of RSV-infected mice. Survival was monitored and recorded for 5 days post-infection (*n* = 8).

Based on the potent neutralizing activity of Hu3F5 *in vitro*, we evaluated its prophylactic efficacy against RSV infection in a cyclophosphamide-induced immunosuppressed mouse model challenged with the RSV A2 strain. Hu3F5 was compared with the control antibody nirsevimab at three weight-based doses (low: 0.5 mg/kg; medium: 2 mg/kg; high: 8 mg/kg). Animals received a single intravenous injection of the respective antibodies 2 h before challenge, while the vehicle control group received PBS. All mice were anesthetized and infected intranasally with 1 × 10⁵ PFU of RSV A2. Lung tissues were harvested 5 days post-infection for viral titer determination (Fig. 3A). Virus titration assays demonstrated that both Hu3F5 and nirsevimab, even at the lowest dose (0.5 mg/kg), significantly reduced viral loads in the lungs of RSV-infected mice compared to the PBS vehicle control group (*P* < 0.0001), effectively suppressing RSV-A2 infection in the lower respiratory tract (Fig. 3B). Notably, Hu3F5 was more potent than nirsevimab in inhibiting viral replication at 0.5 mg/kg, clearing viral titers to undetectable levels (*P* = 0.0025; Fig. 3B). Furthermore, all treatment groups exhibited 100% survival and maintained stable body weight throughout the study (Fig. 3C and D), demonstrating that Hu3F5 effectively protected mice against RSV A2 infection.

### Impact of RSV F protein mutations at site Ø on Hu3F5 binding activity

**Fig 4:**
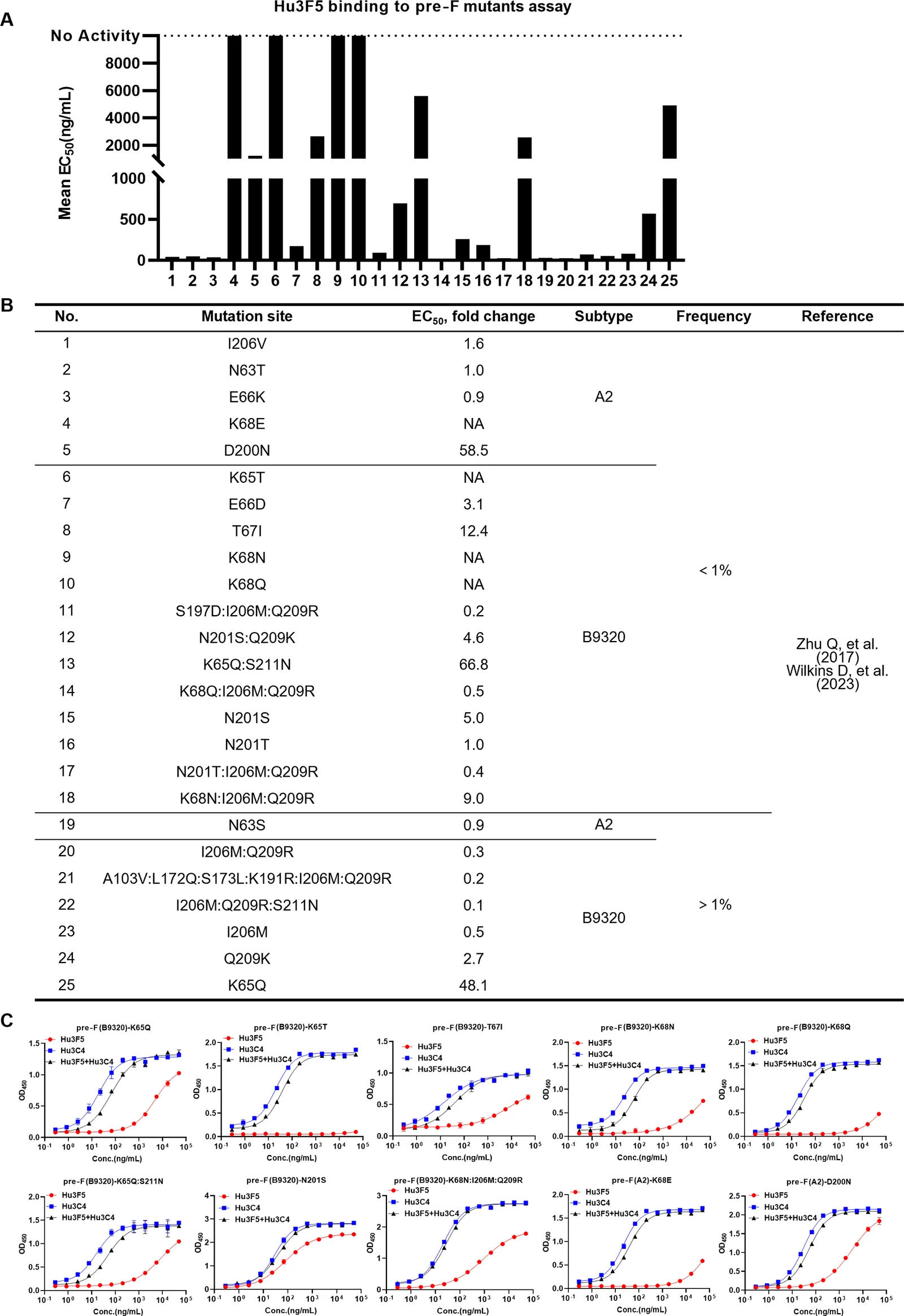
The combination of Hu3F5 and Hu3C4 demonstrates potential for broad-spectrum neutralization. (**A**) Mean ELISA EC_50_ values of Hu3F5 binding to prevalent pre-F mutations at the Ø epitope (*n* = 2). The dashed line indicates loss of binding activity of Hu3F5 to the pre-F mutant. Data are representative of one of three independent experiments. (**B**) Summary of mutations at the Ø epitope of F protein, their occurrence frequency, and changes in binding activity of Hu3F5. Fold change was calculated by dividing the EC_50_ of variant F proteins by the EC_50_ of wild-type F protein. NA, not available. (**C**) ELISA analysis of Hu3F5, Hu3C4, and their combination binding to recombinant pre-F mutants (fold change > 5%). Data are representative of one of three independent experiments and presented as mean ± SD (*n* = 2). ELISA, enzyme-linked immunosorbent assay.

Despite the high conservation of the RSV F protein, immune escape mutations can occur. To evaluate the impact of RSV F protein variations on the binding activities of Hu3F5, we generated 25 RSV F variants harboring mutations at site Ø reported up to 2023 and assessed their binding via ELISA. Hu3F5 demonstrated broad binding coverage against this panel of antigenic site Ø variants, with the exception of mutations at residues K65 and K68, which abrogated Hu3F5 binding activity (Fig. 4A and B). However, it is noteworthy that the clinical prevalence of these two mutations is very low (<1%) (24).

To address the loss of Hu3F5 efficacy caused by RSV F protein mutations, we employed Hu3C4, which targets an epitope distinct from that of Hu3F5, to complement its activity. We evaluated the effect of the combination by ELISA against variants that exhibited a >5% reduction in binding activity. The results demonstrated that the combination of Hu3F5 and Hu3C4 effectively restored binding. This finding also indicated that the combined use of Hu3F5 and Hu3C4 showed high potential for achieving broad-spectrum neutralization (Fig. 4C).

### Hu3F5 is well tolerated in cynomolgus monkeys up to 500 mg/kg

**Fig 5:**
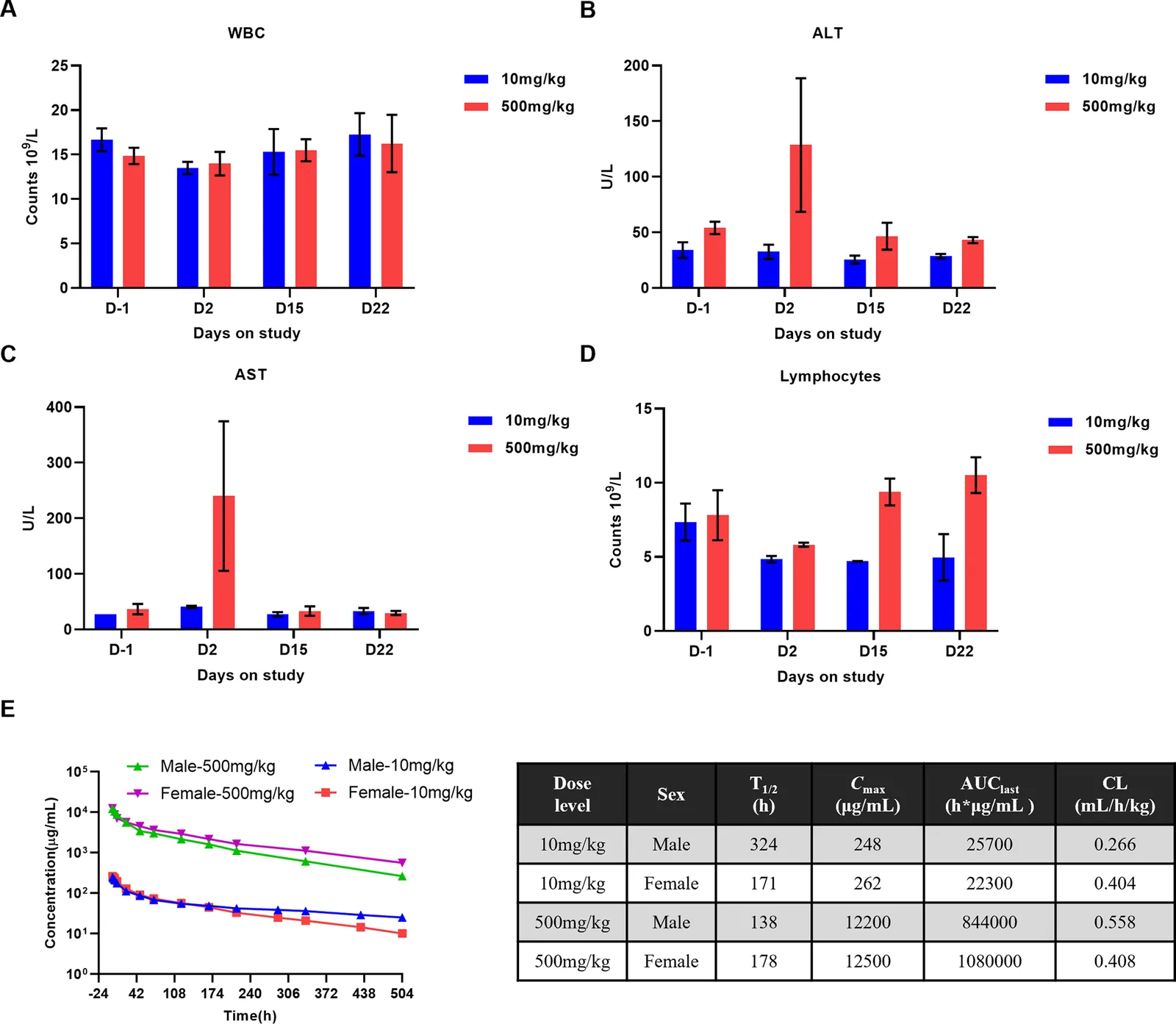
Hu3F5 is well tolerated in cynomolgus monkeys up to 500 mg/kg. (**A**) Cynomolgus monkeys received Hu3F5 via slow intravenous infusion at 10 mg/kg (*n* = 2) and 500 mg/kg (*n* = 2). Blood samples were collected at the indicated time points. Biomarkers, assessed from day −1 to day 22 post-dosing, included leukocyte counts (**A**), liver injury markers (ALT and AST) (**B, C**), and Lymphocyte Counts (**D**). (**E**) The concentration of Hu3F5 in blood was detected by ELISA to determine the PK parameters presented in the table. ELISA, enzyme-linked immunosorbent assay; PK, pharmacokinetic.

To evaluate the safety of Hu3F5, we conducted a single-dose tolerability study in cynomolgus monkeys and analyzed its pharmacokinetic (PK) profiles. No significant Hu3F5-related changes were detected in clinical observations (body weight and temperature) or in white blood cell counts (Fig. 5A). Additionally, elevations in liver enzymes (alanine aminotransferase [ALT] and aspartate aminotransferase [AST]) were noted on day 2 but were reversible by day 15 (Fig. 5B and C). A reduction in lymphocyte counts was observed on day 2; however, this effect was also present in the low-dose (10 mg/kg) group, suggesting no dose dependency. Furthermore, this transient reduction had recovered by day 15 (Fig. 5D). Collectively, these findings indicate that Hu3F5 was well tolerated at high doses in cynomolgus monkeys.

The PK profile of Hu3F5 was characterized in both dose groups, and the results were consistent with expectations. Systemic exposure was dose-related and remained detectable until the last measured time point (day 21). Serum concentration–time profiles were generally consistent between male and female animals within the same dose group (Fig. 5E).

### Hu3F5 exerts its mechanism of action independently of Fc domain function

To mitigate the risk of antibody-dependent enhancement (ADE) associated with Fc-mediated effector functions, we engineered a humanized Fc-fusion protein (Hu3F5) containing L234A/L235A (LALA) mutations in the IgG1 Fc domain (27). These mutations are known to ablate FcγR binding and consequently abolish ADCC activity (28) without compromising antigen-binding affinity (29). Consistent with this, ELISA demonstrated that Hu3F5 retained binding activity to pre-F, comparable to the unmutated llama-derived 3F5 (Fig. 1A). Critically, BLI assays confirmed that Hu3F5 exhibited no detectable binding to key FcγRs, including CD16a, CD64, CD32b, and CD32a (Fig. S6). Collectively, these results indicate that the neutralizing activity of Hu3F5 is solely mediated by its antigen-binding domain and is independent of Fc-mediated effector functions.

### Molecular determinants of Hu3F5 binding on prefusion F protein

**Fig 6:**
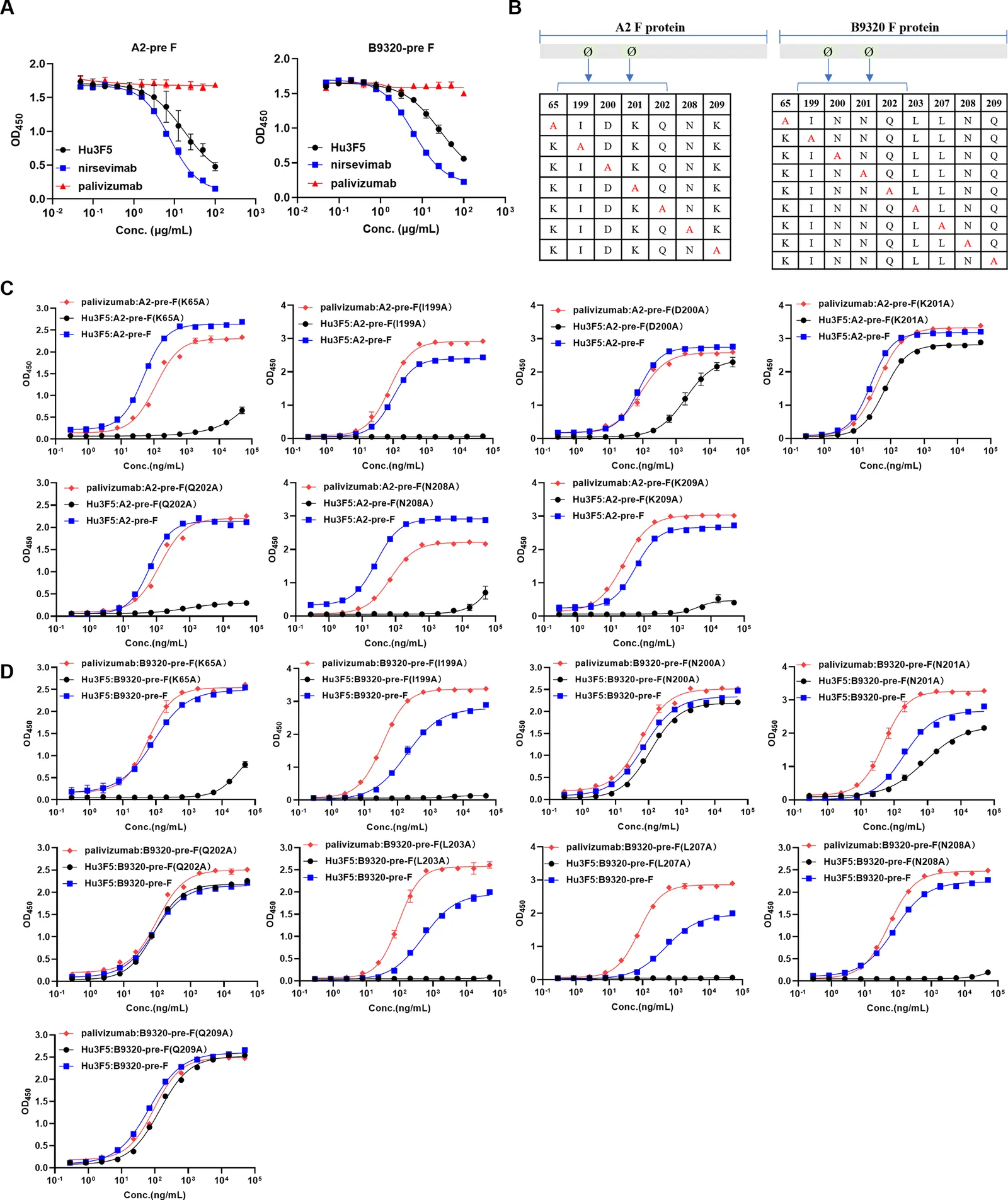
Molecular determinants of Hu3F5 binding on prefusion F protein. (**A**) Competition between Hu3F5 and nirsevimab for binding to A2-pre-F and B9320-pre-F was determined by ELISA. The nirsevimab-binding signal was detected by a corresponding secondary antibody. Data are mean  ±  SD of duplicate wells of one of three independent experiments. (**B**) Schematic diagram of the representative alanine scanning mutagenesis strategy for the Ø epitope on the F protein. (**C and D**) ELISA analysis of Hu3F5 binding to mutated pre-F proteins. Data from one of three independent experiments (*n* = 2) are shown. Palivizumab was used as a positive control. ELISA, enzyme-linked immunosorbent assay.

We found that Hu3F5 blocks RSV entry through direct neutralization independent of Fc-mediated effector functions. Therefore, we sought to identify the key amino acid residues critical for its binding to the F protein of both A2 and B9320 subtypes. Hu3F5’s specificity for the pre-F conformation suggested a conformation-dependent epitope, which was confirmed to overlap with the “Ø” epitope of nirsevimab by both BLI and competitive ELISA (Fig. 2D and 6A). We therefore generated a series of alanine-substitution mutants at key nirsevimab-binding residues (30) and adjacent sites (Fig. 6B). The binding activity of Hu3F5 to each mutant was evaluated by ELISA, with the proper folding and reactivity of these F protein mutants confirmed using palivizumab, a control antibody that does not bind the Ø epitope. We conclude that the following residues are critical for Hu3F5 binding: K65, K68, I199, Q202, N208, and K209 for binding to RSV A2 F (Fig. 4B and 6C) and K65, K68, I199, L203, N207, and K208 for binding to RSV B9320 F (Fig. 4B and 6D).

### Hu3F5 locks the F protein in prefusion conformation to prevent membrane fusion

**Fig 7:**
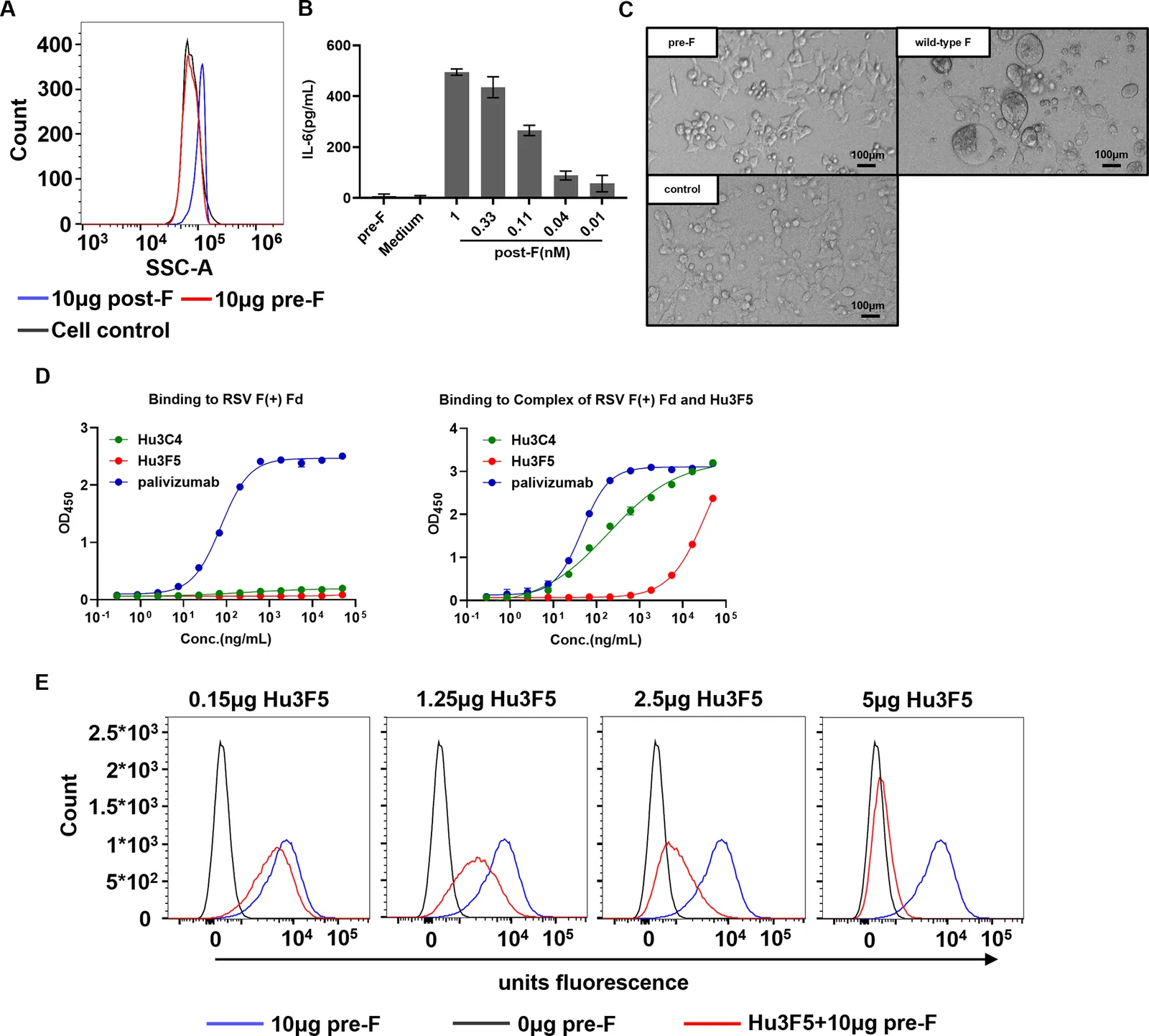
Mechanism of Hu3F5-mediated locking of F protein in prefusion conformation. (**A**) Post-F co-incubation with A549 cells induces a right shift in SSC signal (higher complexity) over background (black), indicating increased internal cellular complexity, as detected by flow cytometry. Pre-F served as a negative control. Data are representative of one of three independent experiments. (**B**) Mouse peritoneal macrophages were stimulated with various concentrations of post-F protein or with pre-F protein (1 nM). Control cultures were treated with medium alone. Supernatants were collected 18 h post-stimulation, and IL-6 concentrations were measured by ELISA. Data are presented as mean ± SD (*n* = 2). Data are representative of one of three independent experiments (**A and B**). (**C**) Syncytium formation induced by transient expression of the RSV wild-type F protein in 293T cells. No syncytia were observed in cells expressing the pre-F protein or in the control cells. Scale bar is 100 μm. (**D**) ELISA-based conformational analysis of F protein in purified products from transfection with RSV F(+) Fd alone or co-transfection with RSV F(+) Fd and Hu3F5, utilizing conformation-specific binding properties. Hu3C4 served as a positive control specific for the pre-F conformation, while palivizumab was used as a positive control binding to both pre-F and post-F conformations. Data are representative of one of three independent experiments (*n* = 3). (**E**) Hu3F5 blocks pre-F binding to A549 cells as detected by flow cytometry. A left shift of the A549 population (reduced signal intensity) relative to the pre-F-only group (blue) indicates decreased binding of Bio-pre-F to the cells. Data are representative of one of three independent experiments. ELISA, enzyme-linked immunosorbent assay.

To evaluate the cellular effects induced by different conformations of the F protein, we first analyzed signal changes following co-incubation of the two F protein conformations with A549 cells using flow cytometry. Post-F treatment resulted in a significant increase in SSC-A signal, whereas the pre-F group exhibited signals comparable to those of the untreated group (Fig. 7A), demonstrating that post-F treatment significantly altered cellular morphology. Given that the F protein induces the secretion of inflammatory cytokines such as IL-6 from monocytes and contributes to cytopathic syncytia formation (13, 16), we sought to determine whether the F protein conformation leads to differential inflammatory responses and cytopathic effects. We measured IL-6 levels in the supernatants of mouse peritoneal macrophages stimulated with pre-F or post-F protein. Stimulation with post-F protein induced significant IL-6 secretion, whereas treatment with pre-F protein did not elicit IL-6 production (Fig. 7B). Furthermore, we demonstrated that the expression of the wild-type F protein on the cell surface led to cytopathic syncytia formation, whereas this cytopathic effect was not observed in cells expressing the prefusion-stabilized F protein (Fig. 7C). Based on these findings, we hypothesized that Hu3F5 neutralizes RSV by locking the F protein in its prefusion conformation, thereby blocking the RSV-induced infection response.

To evaluate the ability of Hu3F5 to lock the conformation of the RSV F protein, we co-transfected 293F cells with Hu3F5 and a construct encoding the wild-type F protein ectodomain fused to a C-terminal fibritin trimerization domain, containing no stabilizing mutations (designated RSV F(+) Fd) (11). Transfection with RSV F(+) Fd alone served as the control. Based on the distinct binding profiles of conformation-specific antibodies, we compared the conformation of RSV F(+) Fd expressed in the presence or absence of Hu3F5 by ELISA. The results showed that palivizumab could bind both the RSV F(+) Fd-Hu3F5 complex and RSV F(+) Fd alone. Notably, Hu3C4, which preferentially recognizes pre-F at an epitope distinct from Hu3F5, could efficiently bind the RSV F(+) Fd-Hu3F5 complex but showed no binding activity to RSV F(+) Fd alone. As expected, Hu3F5 itself demonstrated minimal binding to the pre-formed complex due to epitope occupancy (Fig. 7D). The binding profile of palivizumab, which targets antigenic site II present in both pre-F and post-F (31, 32), confirmed the presence of F protein in both samples. Furthermore, the conformation-specific binding of Hu3C4 (pre-F preferential) to the RSV F(+) Fd-Hu3F5 complex provides direct evidence that Hu3F5 binding effectively locks the F protein ectodomain in the prefusion conformation.

To further investigate the effect of Hu3F5 on F protein binding to the cell membrane, we analyzed the interaction of pre-F with cell membranes following Hu3F5 treatment using flow cytometry. Hu3F5 significantly inhibited F protein binding to cells (Fig. 7E). Collectively, these results demonstrate that Hu3F5 blocks RSV infection by locking the F protein in its prefusion conformation.

## DISCUSSION

Passive immunization has been proven effective in protecting infants and young children from respiratory syncytial virus (RSV) infection, and prophylactic antibody therapy can circumvent the risk of vaccine-enhanced respiratory disease (33, 34). To address the continuing need for effective RSV prophylaxis, we developed Hu3F5, a single-domain antibody-Fc fusion protein targeting the RSV prefusion F protein. The VHH–Fc fusion protein format has recently emerged as a promising antiviral strategy (35), combining the high affinity and favorable biophysical properties of the minimal VHH domain with a substantially smaller molecular size than conventional IgG antibodies (36, 37). In the present study, Hu3F5 exhibited potent neutralizing activity *in vitro* together with robust prophylactic efficacy *in vivo*, supporting the feasibility of this molecular format for RSV passive immunoprophylaxis.

In the cyclophosphamide-induced immunosuppressed mouse model, Hu3F5 significantly reduced pulmonary viral loads following RSV A2 challenge. Under the experimental conditions evaluated, Hu3F5 provided a level of protection comparable to that of the clinically approved monoclonal antibody nirsevimab across all tested dose groups. Notably, at the lowest dose tested (0.5 mg/kg), Hu3F5 reduced pulmonary viral titers to below the limit of detection, whereas residual virus remained detectable in the nirsevimab-treated group. Although this observation should be interpreted cautiously given the limitations of the experimental model and study design, it suggests that Hu3F5 retains potent antiviral activity even under dose-limited conditions. Together with its strong *in vitro* neutralizing activity, these findings further support Hu3F5 as a promising candidate for RSV passive immunoprophylaxis.

The process of RSV infection remains incompletely understood. Current research indicates that the F protein plays a critical role in RSV infection, driving inflammatory responses and the formation of syncytia as cytopathic effects (13, 16). Upon initial contact with the cell membrane during infection, the F protein undergoes conformational changes, suggesting that the two conformational states of the F protein may possess distinct functions. Based on this consideration, we conducted a comparative study of the two F protein conformations at the cellular level. Our results reveal fundamental differences between the prefusion and postfusion conformations of the RSV F protein. The prefusion F protein binds to the cell membrane without inducing detectable cellular changes, whereas the postfusion F protein induces a significant increase in cellular complexity. This disparity can be explained by the exposure of the fusion peptide—a key mediator of membrane fusion—in the postfusion F state. In the prefusion F conformation, the fusion peptide is sequestered within a protected hydrophobic pocket. Upon triggering the transition to the postfusion F state, however, large-scale structural rearrangements eject the fusion peptide to the apex of the trimeric F protein, enabling its insertion into the host cell membrane (38, 39). During RSV entry, F protein binding to the IGF1R receptor activates host protein kinase C zeta (PKCζ), a primary kinase in the viral entry cascade (40–42). PKCζ activation, which involves a series of phosphorylation events (43), promotes the translocation of nucleolin (NCL) from the nucleus to the cell membrane (42, 44). This NCL trafficking event requires the engagement of intracellular transport systems, resulting in cytoskeletal reorganization and a subsequent remodeling of subcellular architecture. Collectively, these intracellular events may contribute to the increased cellular complexity observed following postfusion F stimulation. Furthermore, our results confirmed that postfusion F acts as the major trigger of RSV-induced inflammation, inducing the secretion of inflammatory cytokines from monocytes. More importantly, we demonstrated that only the wild-type F induced the formation of cytopathic syncytia, whereas prefusion F treatment under the same conditions elicited minimal inflammatory response or cytopathic effect. This suggests that conformational rearrangement of the F protein on the RSV surface is a critical step for productive infection.

These mechanistic findings further provide a rationale for the superior efficacy of antibodies targeting the prefusion F protein. Previous studies have shown that antibodies specifically targeting the prefusion F protein are generally more effective than those binding other forms (25). By stabilizing the metastable prefusion conformation, such antibodies are thought to prevent the structural rearrangements required for membrane fusion, syncytium formation, and the initiation of downstream inflammatory responses. Consistent with this model, our co-transfection assays, conformation-specific ELISA, flow cytometric analyses, epitope binning, and alanine-scanning mutagenesis collectively suggest that Hu3F5 targets the prefusion F protein and functionally stabilizes its prefusion conformation, thereby preventing the conformational transition required for viral entry. Although these complementary findings support a proposed “locking” mechanism, direct high-resolution structural evidence for Hu3F5-mediated prefusion stabilization is currently lacking. Therefore, this mechanism should be regarded as a functional inference rather than a structurally validated conclusion. Future high-resolution structural characterization of the Hu3F5–prefusion F complex, for example, by cryo-electron microscopy or X-ray crystallography, will be important for defining the molecular interactions underlying this stabilization mechanism.

In addition to its potent antiviral activity, Hu3F5 exhibited favorable developability characteristics. It retained binding activity against the majority of F protein mutants examined, with loss of activity limited to substitutions at residues K65 and K68, both of which occur at frequencies below 1%. To address this potential vulnerability, we evaluated a combination strategy using Hu3F5 together with Hu3C4, which restored neutralizing activity against all tested variants. This finding suggests a feasible strategy for mitigating the potential emergence of future antigenic mutations. Furthermore, toxicological evaluation in cynomolgus monkeys demonstrated a favorable safety profile, supporting the continued preclinical development of Hu3F5.

Despite these encouraging findings, several limitations should be acknowledged. Although the mouse model provides a widely used experimental platform for evaluating antibody-mediated protection, it does not fully recapitulate the viral replication kinetics or inflammatory pathology observed in human RSV infection (45). Future studies should therefore validate the efficacy and immunomodulatory activity of Hu3F5 in cotton rats, which more faithfully reproduce the virological and pathological characteristics of human disease (46). In addition, the *in vivo* efficacy of Hu3F5 was evaluated only in a prophylactic mouse model using the RSV A2 strain. Although live-virus neutralization assays demonstrated potent activity against representative RSV-A (A2) and RSV-B (B9320 and B18537) strains, *in vivo* validation against RSV-B was not included in the current study. Future studies should also investigate the antiviral efficacy of Hu3F5 following post-exposure administration and evaluate its protective activity against a broader range of clinically relevant RSV isolates, thereby further defining its therapeutic window and translational potential.

In summary, our study identifies Hu3F5 as a potent prefusion F-targeting single-domain antibody-Fc fusion protein with broad neutralizing activity, favorable safety, and robust prophylactic efficacy. More importantly, our findings provide new functional insights into the distinct biological roles of the prefusion and postfusion conformations of the RSV F protein and support stabilization of the prefusion F protein as an effective strategy for RSV passive immunoprophylaxis.

## MATERIALS AND METHODS

### Expression and purification of F proteins

The plasmid constructs encoding pre-F (UniProt: C3UPB8), post-F (UniProt: P03420), A2-pre-F (UniProt: P03420), B9320-pre-F (UniProt: Q6V2E7), and B18537-pre-F (UniProt: P13843) were generated according to previously described methods (47, 48). The constructed plasmids were transiently transfected into Expi293F (Thermo Fisher) cells for protein expression. Cell culture supernatants were harvested on day 6 post-transfection, followed by centrifugation to remove debris and sterile filtration. Proteins were affinity-purified using Ni-NTA Agarose. Pre-F mutants were expressed and purified as described previously.

### Alpaca immunization and pre-F-specific phage screening

The pre-F-specific single-domain antibody (VHH) library was generated following established methodologies (49, 50). Briefly, a recombinant pre-F antigen was used to immunize an alpaca for the generation of the phage display library. The primary immunization was administered via multiple subcutaneous injections with 1 mg of antigen emulsified 1:1 with adjuvant. This was followed by three booster immunizations with 500 μg antigen each. Following the immunization regimen, peripheral blood was collected, and peripheral blood mononuclear cells (PBMCs) were isolated using lymphocyte separation medium. Total RNA was extracted from PBMCs and reverse-transcribed into cDNA. The VHH genes were amplified by nested PCR, cloned into the pMES4 vector, and electroporated into TG1 competent cells to construct the phage display library. After two rounds of biopanning, individual phage clones were isolated and screened by ELISA for high-affinity binding to the pre-F protein, followed by sequencing of selected clones.

### Expression and purification of single-domain antibody-Fc fusion proteins and control antibodies

Single-domain antibody-Fc fusion proteins and control antibodies (nirsevimab and palivizumab) were expressed in Expi293F cells by transient transfection with the respective expression plasmids. Transfected cells were cultured at 37°C with 8% CO_2_ in a humidified incubator. The culture supernatants were harvested on day 7 post-transfection for purification via Protein A affinity chromatography (Cytiva MabSelect SuRe).

### Enzyme-linked immunosorbent assay (ELISA)

Ninety-six-well plates were coated overnight at 4°C with recombinant antigens (0.1 μg/well for pre-F/post-F, 0.2 μg/well for Ø-epitope mutants), blocked with PBST containing 1% BSA for 2 h at room temperature (RT), and then incubated with serially diluted antibodies or Fc-fusion proteins. For competition ELISA, Hu3F5 was serially diluted in a solution containing biotinylated nirsevimab (0.5 μg/mL) and pre-incubated for 1 h before addition to the plates; binding was detected with HRP-streptavidin (1:2,000). For direct binding, detection was performed with HRP-conjugated goat anti-human IgG Fc antibody (1:50,000). After TMB substrate development, reactions were stopped with 1 M H₃PO₄, and the absorbance was read at 450 nm. Half-maximal effective concentrations (EC₅₀) were calculated by nonlinear regression using GraphPad Prism 9.5.

### Bio-layer interferometry (BLI) assay

The BLI assay was conducted using an Octet Red 384 system (Sartorius) to determine the binding affinity of Fc-fusion proteins for various pre-F proteins. Briefly, the Fc-fusion proteins were immobilized on pre-wet Protein A biosensors (Sartorius) at 5 μg/mL. Subsequently, two-fold serial dilutions of pre-F proteins (ranging from 100 nM to 1.56 nM) were flowed over the captured surface for association, followed by dissociation in buffer. The assay was performed in PBST (PBS containing 0.05% Tween-20). The binding kinetics, including the association rate constant (*K*_on_), dissociation rate constant (*K*_off_), and equilibrium dissociation constant (*K*_*D*_), were analyzed by global fitting with a 1:1 binding model using the Octet Analysis Studio 12.1 software.

### Epitope binning of Fc-fusion proteins

Epitope binning was performed on the Octet RED384 system. Briefly, pre-F antigen (30 nM) was immobilized onto Ni-NTA biosensors (Sartorius) with a loading threshold of 0.6 nm. The sensors were then incubated with the first Fc-fusion protein (250 nM) for association. Subsequently, the resulting antigen–Fc-fusion complex was exposed to the second Fc-fusion protein (250 nM). The binning assay included two reciprocal cycles for validation, with self-competition serving as the control. Data were processed and analyzed using the Octet Analysis Studio software according to the manufacturer’s protocol.

### RSV neutralization assay

HEp-2 cells (ATCC) were seeded in 96-well plates at a density of 2 × 10⁴ cells per well and incubated overnight. The next day, Fc-fusion proteins (starting at 20 μg/mL with serial 4-fold dilutions) were pre-mixed with RSV (2 × 10⁴ PFU/mL) for 1 h at 37°C. An equal-volume mixture of the viral solution and dilution buffer served as the virus control (VC), while dilution buffer alone served as the cell control (CC). The mixtures were then added to the HEp-2 cells in duplicate. Following a 2-h infection period, after replacing the medium with 2% FBS-DMEM, cells were incubated for 21–22 h, fixed, and blocked.

Viral infection was detected using the F6-6-GFP antibody (Vazyme; a recombinant human IgG1 specific for a linear epitope of the RSV G protein and conjugated with GFP) and quantified via an ImmunoSpot analyzer (CTL). The inhibition rate was calculated as follows: Inhibition rate = 1 − [(sample − CC) / (VC − CC)]. The half-maximal inhibitory concentration (IC₅₀) was determined by nonlinear regression analysis using GraphPad Prism 9.5.

### *In vivo* assessment of Hu3F5 prophylactic efficacy against RSV A2 infection

Female BALB/c mice (6–8 weeks old) were immunosuppressed with cyclophosphamide (100 mg/kg, i.p.) on days −6 and −1 relative to RSV challenge. On day 0, mice (*n* = 8 per group) received a single intravenous injection of Hu3F5 or nirsevimab at three dosage levels (8 mg/kg, 2 mg/kg, and 0.5 mg/kg). PBS was used as the vehicle control. Two hours post-injection, all animals were anesthetized and intranasally inoculated with 1 × 10⁵ PFU of RSV A2 strain. Body weight and survival were monitored daily. On day 5 post-infection, mice were euthanized, and lungs were harvested for viral titer determination by plaque assay. Lung tissues were homogenized in HBSS (10% wt/vol), and the homogenates were inoculated onto replicate HEp-2 monolayers at different volumes. Following a 5-day incubation at 37°C under 5% CO₂, the cells were fixed with 10% formalin and stained with crystal violet. Plaques were then counted.

### Cynomolgus monkey safety and pharmacokinetic (PK) study

Four cynomolgus monkeys received a single slow intravenous infusion of Hu3F5 at doses of 10 mg/kg (*n* = 2) and 500 mg/kg (*n* = 2), with each dose group comprising one male and one female animal. Blood samples were collected at various time points for pharmacokinetic (PK) assessment, and clinical observations were performed at designated times. Serum concentrations of Hu3F5 were quantified using a sandwich ELISA. PK parameters were derived by non-compartmental analysis using Phoenix WinNonlin (version 8.1).

### Flow cytometry assay

A549 cells (Cell Bank of Chinese Academy of Sciences) were blocked with PBS containing 5% fetal bovine serum (FBS) on ice for 30 min. Subsequently, the cells were incubated with a pre-mixed complex of biotinylated pre-F-Hu3F5 on ice for 30 min. After washing with PBS containing 1% FBS, the cells were stained with PE streptavidin (BioLegend, 405204) under dark conditions on ice for 30 min. Finally, the cells were analyzed on a flow cytometer (BD FACSVerse), and the data were processed with FlowJo 10.8.1.

### Assessment of IL-6 secretion *in vitro*

Mouse peritoneal macrophages (1 × 10⁵ cells/well) were cultured with serial dilutions of the F protein for 18 h. Supernatants were then collected and analyzed for cytokine levels by ELISA.

### F protein mediated cell-cell fusion

The syncytium formation assay was performed as previously described (13). To evaluate fusion activity, HEK293T cells (Cell Bank of the Chinese Academy of Sciences) were transiently transfected with a pcDNA3.4 expression vector encoding a codon-optimized full-length wild-type RSV F gene (residues 1–568). Cell-surface expression of the prefusion-stabilized F (pre-F) variant was achieved by transfecting cells with a pcDNA3.4 plasmid encoding a codon-optimized RSV F gene harboring the cavity-filling and disulfide-locking mutations (S155C, S190F, V207L, and S290C) (51). At 24 h post-transfection, cells from each transfection group (2.5 × 10⁴ cells/well) were added to wells pre-seeded with 5 × 10⁴ untransfected 293T cells. The co-cultured cells were then incubated at 37°C for an additional 24 h, after which cell morphology was observed and photographed.

### Conformational analysis of F protein in different transfection conditions

Wild-type respiratory syncytial virus (RSV) F protein (residues 1–513) fused to a C-terminal fibritin trimerization domain (RSV F(+) Fd) with a 6 × His tag was expressed in Expi293F cells by transient transfection. At 4 h post-transfection, one culture was supplemented with Hu3F5 (50 μg/mL), while the other served as an untreated control. Seven days post-transfection, proteins were purified from the culture supernatant by Ni-NTA affinity chromatography. The conformations of purified proteins from both conditions were determined by ELISA.

### Statistical analysis

All experiments were performed with at least three replicates unless otherwise specified. Statistical analysis of ELISA data was conducted using Prism software. For *in vivo* pharmacodynamic assessments, statistical significance was determined by one-way ANOVA with Tukey’s multiple comparisons test. Statistical significance was defined as follows: ****, *P* < 0.0001; ***, *P* < 0.001; **, *P* < 0.01; *, *P* < 0.05; ns, *P* > 0.05.

## SUPPLEMENTAL MATERIAL

Fig. S1 aac.00541-26-s0001.docx Characterization of the pre-F protein.

Fig. S2 aac.00541-26-s0002.docx Screening and identification of pre-F-targeting neutralizing single-domain antibody-Fc fusion proteins.

Fig. S3 aac.00541-26-s0003.docx Kinetic binding analysis of 12 Fc-fusion proteins to the pre-F protein.

Fig. S4 aac.00541-26-s0004.docx Screening for candidates with *in vitro* neutralizing activity against live virus.

Fig. S5 aac.00541-26-s0005.docx Comparative binding analysis of five humanized Fc-fusion proteins to three wild-type RSV pre-F proteins.

Fig. S6 aac.00541-26-s0006.docx Hu3F5 exerts its mechanism of action independently of Fc domain function.

Table S1 aac.00541-26-s0007.docx Summary of the BLI affinity values of 12 Fc-fusion proteins for recombinant pre-F.

ASM does not own the copyrights to Supplemental Material that may be linked to, or accessed through, an article. The authors have granted ASM a non-exclusive, world-wide license to publish the Supplemental Material files. Please contact the corresponding author directly for reuse.
