# Supplementary material for: Mechanistic blockade of RSV infection by a pre-F targeting single-domain antibody-Fc fusion protein
Source: Antimicrob Agents Chemother. 2026 Sep 16;70(10):e00541-26. doi: 10.1128/aac.00541-26 (PMC13689173; doi:10.1128/aac.00541-26)
Supplement: Supplementary file 1 [file aac.00541-26-s0001.docx]

**Fig S1. Characterization of the pre F protein. (**A) Size exclusion chromatography profile of the purified pre F protein. (B) Binding of the recombinant pre F protein to Nirsevimab was measured by ELISA. Data are representative of three independent experiments and are shown as the mean ± SD (n=2). HMW, high molecular weight; LMW, low molecular weight; ELISA, enzyme-linked immunosorbent assay.
