# Supplementary material for: Mechanistic blockade of RSV infection by a pre-F targeting single-domain antibody-Fc fusion protein
Source: Antimicrob Agents Chemother. 2026 Sep 16;70(10):e00541-26. doi: 10.1128/aac.00541-26 (PMC13689173; doi:10.1128/aac.00541-26)
Supplement: Supplementary file 2 [file aac.00541-26-s0002.docx]

**Fig S2. Screening and identification of pre F-targeting neutralizing single domain antibody-Fc fusion proteins.** The binding activity of 12 Fc-fusion proteins with recombinant pre F and post F were detected by ELISA. Palivizumab was used as a positive control for binding to post F. Data presents one of three independent experiments. ELISA, enzyme-linked immunosorbent assay; EC_50_, half maximal effective concentration.
