# Supplementary material for: Mechanistic blockade of RSV infection by a pre-F targeting single-domain antibody-Fc fusion protein
Source: Antimicrob Agents Chemother. 2026 Sep 16;70(10):e00541-26. doi: 10.1128/aac.00541-26 (PMC13689173; doi:10.1128/aac.00541-26)
Supplement: Supplementary file 3 [file aac.00541-26-s0003.docx]

**Fig S3. Kinetic binding analysis of 12 Fc-fusion proteins to the pre F protein.** (A-L) Kinetic binding curves of the Fc-fusion proteins as measured by BLI. Data are representative of one of three independent experiments. BLI, bio-layer interferometry.
