# Supplementary material for: Mechanistic blockade of RSV infection by a pre-F targeting single-domain antibody-Fc fusion protein
Source: Antimicrob Agents Chemother. 2026 Sep 16;70(10):e00541-26. doi: 10.1128/aac.00541-26 (PMC13689173; doi:10.1128/aac.00541-26)
Supplement: Supplementary file 4 [file aac.00541-26-s0004.docx]

**Fig S4. Screening for candidates with in vitro neutralizing activity against live virus.** The neutralizing activity of the five Fc-fusion proteins against the RSV Long strain was measured using a live virus neutralization assay. Data presents one of three independent experiments, shown as mean±SD (n=2). Nirsevimab served as a positive control. Data presents one of three independent experiments. IC_50_, half maximal inhibitory concentration.
