# Supplementary material for: Mechanistic blockade of RSV infection by a pre-F targeting single-domain antibody-Fc fusion protein
Source: Antimicrob Agents Chemother. 2026 Sep 16;70(10):e00541-26. doi: 10.1128/aac.00541-26 (PMC13689173; doi:10.1128/aac.00541-26)
Supplement: Supplementary file 5 [file aac.00541-26-s0005.docx]

**Fig S5. Comparative binding analysis of five humanized Fc-fusion proteins to three wild-type RSV pre F proteins.** (A) Binding curves to RSV A2-pre F. (B) Binding curves to RSV B9320-pre F. (C) Binding curves to RSV B18537-pre F. Data are representative of one of three independent experiments and are shown as the mean ± SD (n=2). EC_50_, half maximal effective concentration; ELISA, enzyme-linked immunosorbent assay.
