# Supplementary material for: Mechanistic blockade of RSV infection by a pre-F targeting single-domain antibody-Fc fusion protein
Source: Antimicrob Agents Chemother. 2026 Sep 16;70(10):e00541-26. doi: 10.1128/aac.00541-26 (PMC13689173; doi:10.1128/aac.00541-26)
Supplement: Supplementary file 6 [file aac.00541-26-s0006.docx]

**Fig S6. Hu3F5 exerts its mechanism of action independently of Fc domain function**. (A-D) Binding kinetics of Hu3F5 to Fcγ receptors. Real-time binding and dissociation profiles of Hu3F5 with (A) FcγRIIIa (CD16a), (B) FcγRI (CD64), (C) FcγRIIb (CD32b), and (D) FcγRIIa (CD32a) as measured by bio-layer interferometry (BLI). VHH-Fc-WT was used as positive control. Data are representative of one of three independent experiments (A-D).
