# Supplementary material for: Mechanistic blockade of RSV infection by a pre-F targeting single-domain antibody-Fc fusion protein
Source: Antimicrob Agents Chemother. 2026 Sep 16;70(10):e00541-26. doi: 10.1128/aac.00541-26 (PMC13689173; doi:10.1128/aac.00541-26)
Supplement: Supplementary file 7 [file aac.00541-26-s0007.docx]

**TABLE S1.** Summary of the BLI affinity values of 12 Fc-fusion proteins for recombinant pre F.

| **Clone number** | **K_on_ (1/Ms)** | **K_off_ (1/s)** | **K_D_ (nM)** |
| --- | --- | --- | --- |
| 3F5 | 6.201×10^5^ | 1.805×10^-5^ | 0.0291 |
| 2D1 | 1.502×10^5^ | 3.902×10^-6^ | 0.026 |
| 4H10 | 1.421×10^5^ | 6.488×10^-6^ | 0.0457 |
| 2B2 | 2.298×10^5^ | 3.092×10^-5^ | 0.135 |
| 5C3 | 1.681×10^5^ | 1.387×10^-7^ | <0.001 |
| 3C3 | 1.848×10^5^ | 4.468×10^-5^ | 0.242 |
| 3C4 | 2.889×10^5^ | 3.656×10^-5^ | 0.127 |
| 3B4 | 1.584×10^5^ | 1.346×10^-7^ | <0.001 |
| 1H2 | 1.641×10^5^ | 5.581×10^-5^ | 0.34 |
| 6D1 | 1.217×10^5^ | 7.571×10^-6^ | 0.062 |
| 1F8 | 1.438×10^5^ | 9.395×10^-6^ | 0.065 |
| 1H6 | 1.487×10^5^ | 2.464×10^-6^ | 0.017 |

*BLI, bio-layer interferometry; K_D_, equilibrium dissociation constant; K_on_, association rate values; K_off_, dissociation rate values.
